# Assessing the Impact of Estimated Glucose Disposal Rate (eGDR) on Cognitive Function in Older Adults: A NHANES‐Based Machine Learning Study

**DOI:** 10.1111/cns.70524

**Published:** 2025-07-14

**Authors:** Tianyi Wang, Haochen Jiang, Ruwen Zheng, Chuchu Zhang, Xiumei Ma, Yi Liu

**Affiliations:** ^1^ Heilongjiang University of Chinese Medicine Harbin Heilongjiang China; ^2^ College of Traditional Chinese Medicine Changchun University of Chinese Medicine Changchun Jilin China; ^3^ Institute of Information on Traditional Chinese Medicine Chinese Academy of Chinese Medical Sciences Beijing China; ^4^ Department of Traditional Chinese and Western Medicine, Sichuan Clinical Research Center for Cancer Sichuan Cancer Hospital & Institute, Sichuan Cancer Center, University of Electronic Science and Technology of China Chengdu Sichuan China; ^5^ Department of Traditional Chinese and Western Medicine Shandong Cancer Hospital and Institute, Shandong First Medical University and Shandong Academy of Medical Sciences Jinan Shandong China

**Keywords:** cognitive impairment, cross‐sectional study, eGDR, insulin resistance, machine learning, NHANES

## Abstract

**Objective:**

This study investigates the relationship between estimated glucose disposal rate (eGDR) and cognitive function, with a focus on its potential as a predictive marker for cognitive impairment in older adults. The study also compares eGDR with other insulin resistance (IR) indices, including the triglyceride‐glucose index (TyG), triglyceride‐to‐high‐density lipoprotein cholesterol ratio (TG/HDL‐C), and metabolic score for insulin resistance (METS‐IR).

**Methods:**

Data were obtained from the National Health and Nutrition Examination Survey (NHANES) for participants aged ≥ 60 years. Cognitive function was assessed using the Consortium to Establish a Registry for Alzheimer's Disease (CERAD), Animal Fluency Test (AFT), and Digit Symbol Substitution Test (DSST). Participants were stratified by eGDR quartiles, and multivariable regression models were applied to evaluate the relationship between eGDR and cognitive impairment. Further analyses included interaction tests, restricted cubic splines (RCS), and machine learning models (LASSO, XGBoost, Random Forest), with performance assessed through ROC curves, decision curve analysis (DCA), and SHAP values.

**Results:**

Higher eGDR levels were significantly associated with improved cognitive scores and a reduced risk of cognitive impairment. For each 1‐unit increase in eGDR, cognitive scores improved by 0.095 points, and the odds of cognitive impairment decreased by 7.5%. Quartile analysis revealed the highest eGDR quartile to be associated with better cognitive function when compared with the lowest quartile. Additionally, the eGDR is potentially more predictive of cognitive dysfunction than other infrared indices. The machine learning model confirms the potential clinical utility of the eGDR in predicting cognitive dysfunction.

**Conclusion:**

eGDR may be a reliable and effective predictor of cognitive function and cognitive impairment risk in older adults. The study suggests that higher eGDR levels may serve as a protective factor against cognitive decline, highlighting the potential importance of managing eGDR for cognitive health, particularly in at‐risk populations. Further research, including longitudinal studies and interventions targeting eGDR components, is needed to confirm these findings and explore potential therapeutic strategies.

## Graphical abstract

Higher estimated glucose disposal rate (eGDR) may be linked to improved cognitive function and reduced risk of cognitive impairment in older adults. eGDR outperforms other insulin resistance indices, highlighting its potential as a predictive marker for cognitive health, with machine learning models confirming its clinical utility.

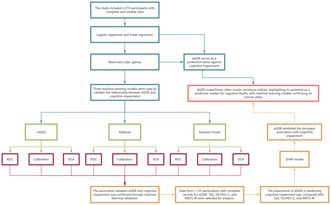

## 1 Introduction

Cognitive ability refers to an individual's capacity to perceive, process, and understand information as well as to respond appropriately, encompassing key domains such as memory, attention, language, and executive function [1]. While mild cognitive decline is a natural aspect of aging, its progression to mild cognitive impairment (MCI) or dementia represents a critical concern [2, 3]. Cognitive impairment is particularly prevalent among individuals aged 65 years and older, with dementia incidence rising significantly after the age of 75 [4, 5, 6]. The global trend of population aging has further exacerbated this issue, with projections indicating a 56% increase in the population aged 60 years and older within the next 15 years, and a doubling of those aged 80 years and older by 2050 [7, 8]. This demographic shift poses substantial challenges, including increased burdens on healthcare systems and caregiving resources, as well as reduced quality of life for affected individuals.

Lifestyle interventions are increasingly recognized as crucial for mitigating cognitive decline [9]. Evidence supports that dietary patterns rich in dark green vegetables, proteins, and antioxidants, along with regular physical activity, are associated with improved cognitive health [10, 11, 12, 13]. Beyond lifestyle factors, several chronic diseases, including diabetes, cardiovascular disease, cancer, and chronic respiratory conditions, are strongly linked to cognitive impairment, often in a dose‐dependent manner [14, 15]. Furthermore, metabolic syndrome (MS)—a constellation of interrelated metabolic abnormalities—has emerged as a significant contributor to neurodegenerative changes and cognitive decline, primarily through insulin resistance (IR), the core pathological mechanism of MS [16, 17, 18].

The euglycemic hyperinsulinemic clamp (EHC), regarded as the gold standard for assessing IR, is not widely utilized in clinical practice due to its complexity, high cost, and the need for specialized expertise [19]. This limitation underscores the need for more accessible and cost‐effective methods to evaluate IR. Several surrogate indices of IR have been proposed, including the estimated glucose disposal rate (eGDR), triglyceride‐glucose index (TyG), triglyceride‐to‐high‐density lipoprotein cholesterol ratio (TG/HDL‐C), and metabolic score for insulin resistance (METS‐IR) [20]. Among these, TyG, TG/HDL‐C, and METS‐IR have been associated with cognitive function in prior studies [21, 22]. The eGDR, calculated using easily obtainable clinical parameters such as glycated hemoglobin (HbA1c), blood pressure, and waist circumference (WC), represents a promising alternative marker for IR that is both cost‐effective and clinically feasible [23, 24]. Previous studies have established associations between eGDR and various conditions, including stroke, infertility, and depression [25, 26, 27]. However, its relationship with cognitive impairment remains underexplored. Moreover, it is unclear whether eGDR demonstrates superior predictive utility for cognitive dysfunction compared to other IR‐related indices, such as TyG, TG/HDL‐C, and METS‐IR. This study aims to fill these critical knowledge gaps by evaluating the association between eGDR and cognitive impairment and comparing its predictive performance with other IR‐related indices.

## 2 Materials and Methods

### 2.1 Date Source and Participants

All data utilized in this study were obtained from the National Health and Nutrition Examination Survey (NHANES), an ongoing research program conducted by the National Center for Health Statistics (NCHS). NHANES integrates interviews and physical examinations to assess the health and nutritional status of a nationally representative sample of approximately 5000 individuals annually, providing critical statistics to the Centers for Disease Control and Prevention (CDC) for public health monitoring and policy development [28]. The research protocol of NHANES was approved by the NCHS Ethics Review Board, and all participants provided written informed consent, with NHANES data being publicly accessible and freely available for secondary analysis [29, 30]. Further details about NHANES can be accessed at https://www.cdc.gov/nchs/nhanes/index.htm.

**FIGURE 1:**
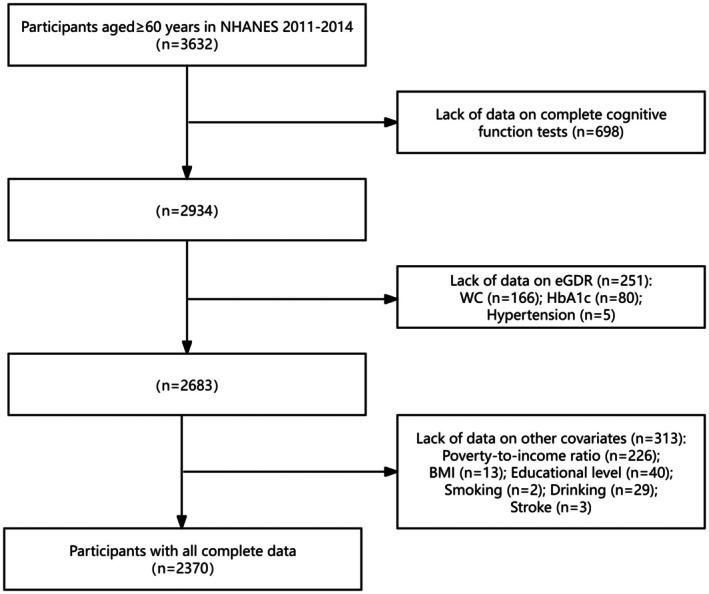
Flowchart of participants in this study.

For this study, we included 3632 participants aged ≥ 60 years who completed cognitive function assessments in the NHANES database from 2011 to 2014. After excluding individuals with incomplete cognitive function assessments and missing data for eGDR and covariates, a total of 2370 participants were retained for analysis. The participant selection process is illustrated in Figure 1.

### 2.2 Cognitive Function Assessment

The cognitive function of participants included in this study was assessed using the Consortium to Establish a Registry for Alzheimer's Disease Word Learning and Recall Module (CERAD), the Animal Fluency Test (AFT), and the Digit Symbol Substitution Test (DSST). The CERAD module consists of three consecutive word learning trials, followed by a delayed word recall test administered after completing the AFT and DSST. Widely utilized in clinical and research settings, CERAD is particularly effective for evaluating cognitive function, with a focus on explicit memory. The CERAD is a comprehensive tool for assessing multiple cognitive domains, including memory, language, visuospatial skills, and executive function, making it particularly valuable for the early diagnosis of dementia by identifying impairments across various cognitive areas [31]. Widely utilized in both clinical and research settings, CERAD is especially effective in evaluating explicit memory, with its Word List Memory Test validated across diverse populations and recognized as a reliable instrument for monitoring cognitive function [32]. The AFT is one of the few neuropsychological assessments that meet the criteria for robust testing and can be fully applied across different linguistic and cultural backgrounds [33]. It evaluates cognitive function by asking participants to name as many animals as possible within 1 min and has proven effective in identifying MCI and Alzheimer's disease (AD) [34]. The DSST is widely used in clinical neuropsychology as an effective and sensitive tool for assessing cognitive impairments, comprehensively measuring processing speed, attention, and executive function by evaluating the speed and accuracy of completing number‐symbol matching tasks within a limited time [35]. The results of the DSST provide critical data for clinicians and researchers to identify abnormal changes in cognitive function and monitor the onset and progression of cognitive impairments [36, 37].

Building on previous studies [38, 39], this research established “Cognitive scores” as the measure of participants' cognitive abilities by integrating their CERAD, AFT, and DSST scores, minimizing discrepancies in assessments across different scoring systems, and controlling for extreme values to reduce biases caused by floor or ceiling effects. $\text{Cognitive scores}=\sum_{1}^{3}\left(x-\text{Mean}\right)/\mathrm{SD}$, *x* represents the score of that test, and mean and SD represent the mean and standard deviation of that test, respectively. Given the absence of a gold standard for diagnosing cognitive impairment through cognitive scores, this study defined cognitive impairment as scores below the 25th percentile (*P*_25_) of “Cognitive scores” (Cognitive scores *P*_25_ = −1.67) [40, 41].

### 2.3 Assessment of eGDR

The equation for eGDR is defined as: $\begin{array}{c} \text{eGDR}=21.158-0.09\times \mathrm{WC} \end{array}$ $\begin{array}{c} \;\left(\mathrm{cm}\right)-3.407\times \mathrm{HP}-0.551\times \mathrm{HbA}1c\;\left(\%\right) \end{array}$ [26], where WC represents waist circumference, HP indicates the presence of hypertension (HP = 1 for hypertensive patients and HP = 0 for non‐hypertensive patients), and HbA1c levels are quantified using high‐performance liquid chromatography, which utilizes ionic interaction‐based separation and photometric detection to measure the stable HbA1c form.

### 2.4 Covariates

The covariates included in this study were gender (male and female), age, race/ethnicity (Mexican American, other Hispanic, Non‐Hispanic White, Non‐Hispanic Black, and other races), educational level (less than high school, high school or GED, and more than high school), poverty‐income ratio (PIR) (PIR > 4 as high income [42], PIR ≤ 4 as non‐high income), and body mass index (BMI) categorized as normal (< 25 kg/m^2^), overweight (25 to < 30 kg/m^2^), and obese (≥ 30 kg/m^2^).

Hypertension was defined as either being diagnosed by a physician or other healthcare professional or currently using antihypertensive medications [43]. Diabetes was defined as being diagnosed by a physician or other healthcare professional, HbA1c levels ≥ 6.5%, fasting plasma glucose (FPG) levels ≥ 7.0 mmol/L after an 8‐h fast, 2‐h plasma glucose levels ≥ 11.1 mmol/L during an oral glucose tolerance test (OGTT), or current use of glucose‐lowering medications or insulin therapy [44]. Stroke was defined as a diagnosis confirmed by a physician or other healthcare professional. Alcohol consumption was categorized into three levels: (1) never: lifetime consumption of < 12 drinks; (2) former: lifetime consumption of ≥ 12 drinks but no drinking in the past year; (3) now: ≥ 12 drinks in the past year. Smoking status was divided into two levels: (1) never: lifetime smoking of ≤ 100 cigarettes; (2) now: lifetime smoking of > 100 cigarettes.

### 2.5 Statistical Analysis

Participants were classified into four groups based on their eGDR levels. Descriptive statistics for continuous variables are presented as mean ± standard deviation (Mean ± SD), while categorical variables are presented as counts and percentages. Group comparisons for categorical variables were performed using the chi‐squared test. For continuous variables, analysis of variance (ANOVA) was used for data that met normality assumptions, while the Kruskal–Wallis test was applied for data that deviated from normality. Detailed results for the normality tests of continuous variables are provided in Table S1. To assess the associations between eGDR levels, eGDR quartiles, and cognitive impairment, multivariable logistic regression models were used. For cognitive scores, multivariable linear regression models were applied. These models were adjusted for potential confounders using three hierarchical approaches: Model 1 (unadjusted), Model 2 (adjusted for age, gender, and race), and Model 3 (adjusted for age, gender, race, education level, income status, alcohol use, smoking status, BMI, and stroke history).

Restricted cubic splines (RCS) were utilized to explore the dose–response relationship between eGDR levels and cognitive impairment. Interaction tests were stratified by age, gender, education level, smoking status, alcohol consumption, stroke history, BMI, and income status. The results were visually presented using forest plots. Additionally, the Boruta algorithm was employed to identify key variables influencing cognitive impairment in this study. Machine learning techniques, including LASSO, XGBoost, and Random Forest, were used to develop and validate predictive models for cognitive impairment based on eGDR levels. The performance of these models was assessed using receiver operating characteristic (ROC) curves and calibration plots, and their clinical utility was further evaluated by decision curve analysis (DCA). Correlation analysis and SHAP (Shapley additive explanations) values were used to assess the relative importance of different IR indices (such as TyG, TG/HDL‐C, and METS‐IR) in predicting cognitive impairment and to identify key factors influencing cognitive health.

All statistical analyses were performed using EmpowerStats (version 4.1) and R software (version 4.3.3). The odds ratio (OR) with 95% confidence intervals (CI) was reported for the relationship between eGDR and cognitive impairment, while *β* coefficients with 95% CI were used to quantify the association between eGDR and cognitive scores. A *p*‐value of less than 0.05 was considered statistically significant.

## 3 Results

### 3.1 Population Characteristics

The study included a total of 2370 participants, who were stratified into four groups based on eGDR quartiles: Q1 (−1.45–4.74), Q2 (4.74–6.18), Q3 (6.18–8.55), and Q4 (8.55–12.37). Significant differences in baseline characteristics were observed across the four groups (*p* < 0.05), as detailed in Table 1. Participants in Q4 were more likely to be high‐income, well‐educated females under the age of 70, with lower rates of stroke and hypertension, higher cognitive scores, and a decreased prevalence of cognitive impairment compared to participants in Q1.

**TABLE 1:** Baseline characteristics of participants (*N* = 2370) by eGDR.

Characteristic	eGDR categories				*p*
	Q1 (−1.45–4.74)	Q2 (4.74–6.18)	Q3 (6.18–8.55)	Q4 (8.55–12.37)	
*N*	593	592	591	594	
Age (years) (%)					< 0.001
< 70	344 (58.01%)	296 (50.00%)	301 (50.93%)	379 (63.80%)	
≥ 70	249 (41.99%)	296 (50.00%)	290 (49.07%)	215 (36.20%)	
Gender (%)					0.014
Male	320 (53.96%)	293 (49.49%)	267 (45.18%)	277 (46.63%)	
Female	273 (46.04%)	299 (50.51%)	324 (54.82%)	317 (53.37%)	
Race (%)					< 0.001
Mexican American	64 (10.79%)	46 (7.77%)	42 (7.11%)	50 (8.42%)	
Other Hispanic	50 (8.43%)	59 (9.97%)	66 (11.17%)	65 (10.94%)	
Non‐Hispanic White	279 (47.05%)	303 (51.18%)	293 (49.58%)	301 (50.67%)	
Non‐Hispanic Black	173 (29.17%)	149 (25.17%)	126 (21.32%)	86 (14.48%)	
Other race	27 (4.55%)	35 (5.91%)	64 (10.83%)	92 (15.49%)	
Education level (%)					< 0.001
Below high school	152 (25.63%)	139 (23.48%)	141 (23.86%)	105 (17.68%)	
High school or GED	151 (25.46%)	147 (24.83%)	146 (24.70%)	117 (19.70%)	
Above high school	290 (48.90%)	306 (51.69%)	304 (51.44%)	372 (62.63%)	
PIR (%)					< 0.001
High income	138 (23.27%)	155 (26.18%)	139 (23.52%)	218 (36.70%)	
Non‐high income	455 (76.73%)	437 (73.82%)	452 (76.48%)	376 (63.30%)	
Alcohol use (%)					0.002
Never	86 (14.50%)	93 (15.71%)	82 (13.87%)	92 (15.49%)	
Former	189 (31.87%)	158 (26.69%)	183 (30.96%)	127 (21.38%)	
Now	318 (53.63%)	341 (57.60%)	326 (55.16%)	375 (63.13%)	
Smoking status (%)					0.005
Never	331 (55.82%)	304 (51.35%)	304 (51.44%)	270 (45.45%)	
Now	262 (44.18%)	288 (48.65%)	287 (48.56%)	324 (54.55%)	
Stroke (%)					0.002
Yes	51 (8.60%)	48 (8.11%)	39 (6.60%)	21 (3.54%)	
No	542 (91.40%)	544 (91.89%)	552 (93.40%)	573 (96.46%)	
Hypertension (%)					< 0.001
Yes	584 (98.48%)	560 (94.59%)	330 (55.84%)	1 (0.17%)	
No	9 (1.52%)	32 (5.41%)	261 (44.16%)	593 (99.83%)	
BMI (%)					< 0.001
Normal	8 (1.35%)	74 (12.50%)	233 (39.42%)	315 (53.03%)	
Overweight	91 (15.35%)	333 (56.25%)	193 (32.66%)	229 (38.55%)	
Obese	494 (83.31%)	185 (31.25%)	165 (27.92%)	50 (8.42%)	
WC (cm)	118.21 ± 11.83	101.84 ± 7.61	97.33 ± 13.77	91.22 ± 9.07	< 0.001
HbA1c (%)	6.78 ± 1.55	6.01 ± 0.96	5.84 ± 0.80	5.67 ± 0.51	< 0.001
CERAD	24.99 ± 6.26	24.95 ± 6.14	24.64 ± 6.73	26.14 ± 6.42	< 0.001
AFT	16.77 ± 5.42	16.26 ± 5.32	16.49 ± 5.43	17.98 ± 5.63	< 0.001
DSST	43.94 ± 16.41	46.07 ± 17.33	46.49 ± 16.84	50.79 ± 16.92	< 0.001
Cognitive scores	−0.22 ± 2.36	−0.19 ± 2.35	−0.17 ± 2.41	0.58 ± 2.38	< 0.001
Cognitive impairment (%)					< 0.001
Yes	158 (26.64%)	162 (27.36%)	171 (28.93%)	104 (17.51%)	
No	435 (73.36%)	430 (72.64%)	420 (71.07%)	490 (82.49%)	

*Note:* Mean ± SD for continuous variable, number (%) for categorical variables.

### 3.2 Relationship Between eGDR and Cognitive

The associations between eGDR levels, its quartiles, and cognitive outcomes were assessed using multiple linear and logistic regression models (Tables 2 and 3). In the fully adjusted Model 3, eGDR was significantly positively associated with cognitive scores (*β* = 0.095, 95% CI: 0.053–0.136, *p* < 0.001), indicating that each 1‐unit increase in eGDR corresponded to a 0.095‐point improvement in cognitive scores. Quartile analysis further demonstrated significantly higher cognitive scores across all quartiles compared to Q1 (*p* < 0.05). The most pronounced increase was observed in Q4, with a 0.541‐point rise in cognitive scores (*β* = 0.541, 95% CI: 0.265–0.816, *p* < 0.001). Similarly, eGDR showed a significant inverse association with the risk of cognitive impairment (OR = 0.925, 95% CI: 0.873–0.980, *p* < 0.01), indicating a 7.5% reduction in the odds of cognitive impairment for each 1‐unit increase in eGDR. Quartile analysis reinforced these findings, with participants in Q4 demonstrating 32.8% lower odds of cognitive impairment compared to those in Q1 (OR = 0.672, 95% CI: 0.457–0.987, *p* < 0.05).

**TABLE 2:** Analyzing the relationship between eGDR and cognitive scores.

	Model 1	Model 2	Model 3
	*β* (95% CI)	*β* (95% CI)	*β* (95% CI)
Cognitive scores			
eGDR continuous	0.118 (0.080, 0.156)***	0.083 (0.049, 0.117)***	**0.095 (0.053, 0.136)**< 0.001
eGDR categories			
Q1	0	0	0
Q2	0.025 (−0.245, 0.295)	0.182 (−0.054, 0.419)	0.242 (0.002, 0.482)*
Q3	0.044 (−0.226, 0.314)	0.136 (−0.101, 0.374)	0.260 (0.007, 0.512)*
Q4	0.802 (0.532, 1.072)***	0.558 (0.319, 0.797)***	**0.541 (0.265, 0.816)**< 0.001

*Note:* Model 1 adjusted for: none. Model 2 adjusted for: age, gender, race. Model 3 adjusted for: age, gender, race, education level, income status (high vs. non‐high), alcohol use, smoking status, BMI, stroke.

* *p* < 0.05.

*** *p* < 0.001.

**TABLE 3:** Analyzing the relationship between eGDR and cognitive impairment.

	Model 1	Model 2	Model 3
	OR (95% CI)	OR (95% CI)	OR (95% CI)
Cognitive impairment			
eGDR continuous	0.929 (0.895, 0.965)***	0.940 (0.902, 0.981)**	**0.925 (0.873, 0.980)**0.008
eGDR categories			
Q1	1	1	1
Q2	1.037 (0.803, 1.341)	0.927 (0.703, 1.223)	0.887 (0.644, 1.222)
Q3	1.121 (0.869, 1.446)	1.078 (0.818, 1.420)	1.003 (0.717, 1.403)
Q4	0.584 (0.442, 0.773)***	0.663 (0.490, 0.895)**	**0.672 (0.457, 0.987)**0.043

*Note:* Model 1 adjusted for: none. Model 2 adjusted for: age, gender, race. Model 3 adjusted for: age, gender, race, education level, income status (high vs. non‐high), alcohol use, smoking status, BMI, stroke.

* *p* < 0.05.

** *p* < 0.01.

*** *p* < 0.001.

**FIGURE 2:**
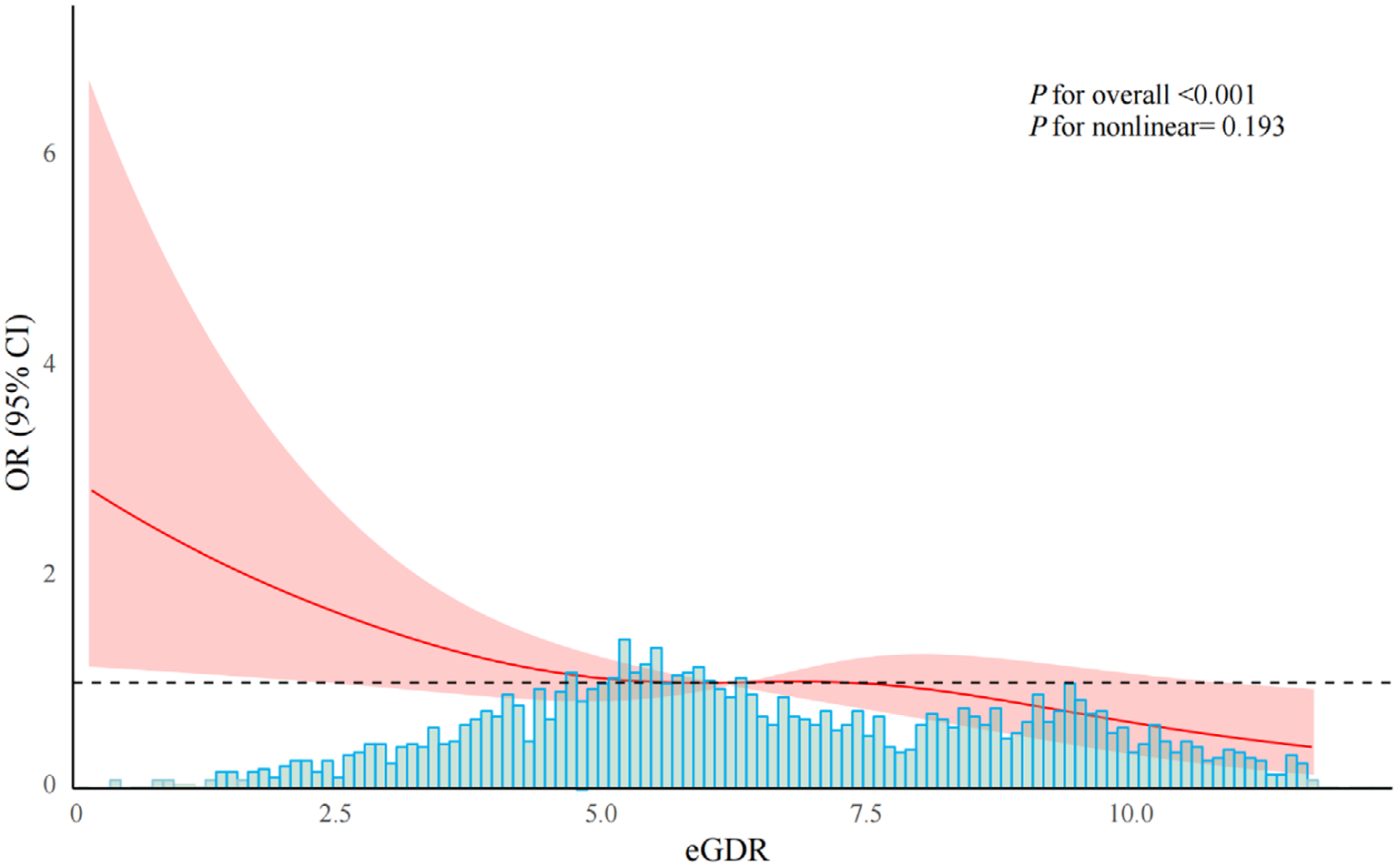
Restricted cubic spline between eGDR and cognitive impairment.

After adjusting for potential confounders, RCS analysis was performed to evaluate the relationship between eGDR levels and the risk of cognitive impairment. As illustrated in Figure 2, eGDR demonstrated a significant linear inverse association with the risk of cognitive impairment (*p* for overall < 0.001), with no evidence supporting a nonlinear relationship (*p* for nonlinearity = 0.139). These results indicate that higher eGDR levels may act as a protective factor against cognitive impairment.

### 3.3 Interaction test

**FIGURE 3:**
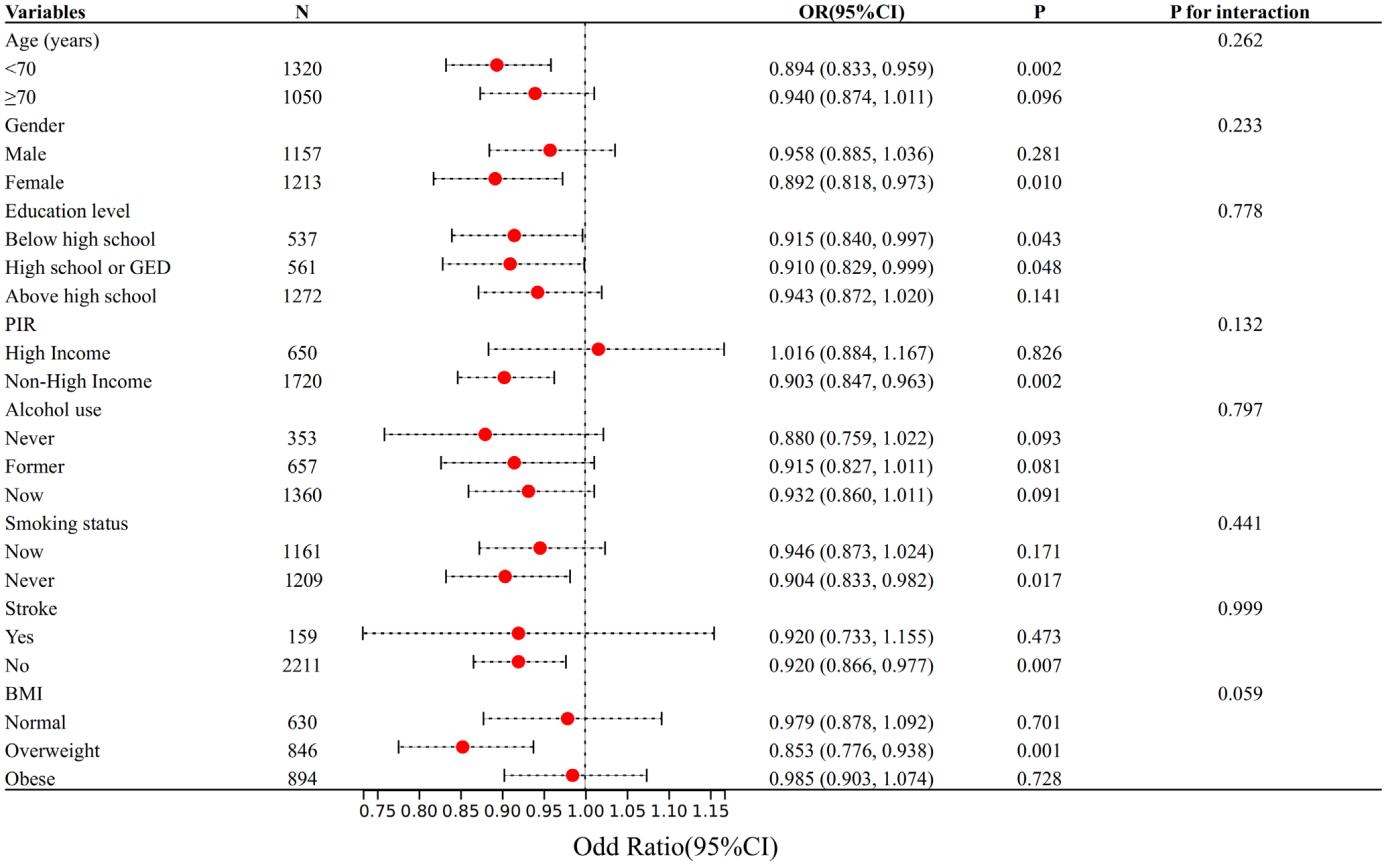
Forest plot of the association between eGDR levels and cognitive impairment across subgroups.

To assess the reliability of the relationship between eGDR and cognitive impairment, interaction tests were conducted for age, gender, education level, income status (high vs. non‐high), alcohol consumption, smoking status, BMI, and history of stroke, as well as for interactions between these factors. The findings (Figure 3) consistently showed a negative association between eGDR and cognitive impairment across all subgroups, with the strongest association observed in overweight women under 70 years old with lower education levels, non‐high income, nonsmoking status, and no history of stroke (*p* < 0.05). No significant interactions were detected across subgroups (*p* for interaction > 0.05).

### 3.4 Identification of Key Variables for Cognitive Impairment Using the Boruta Algorithm

**FIGURE 4:**
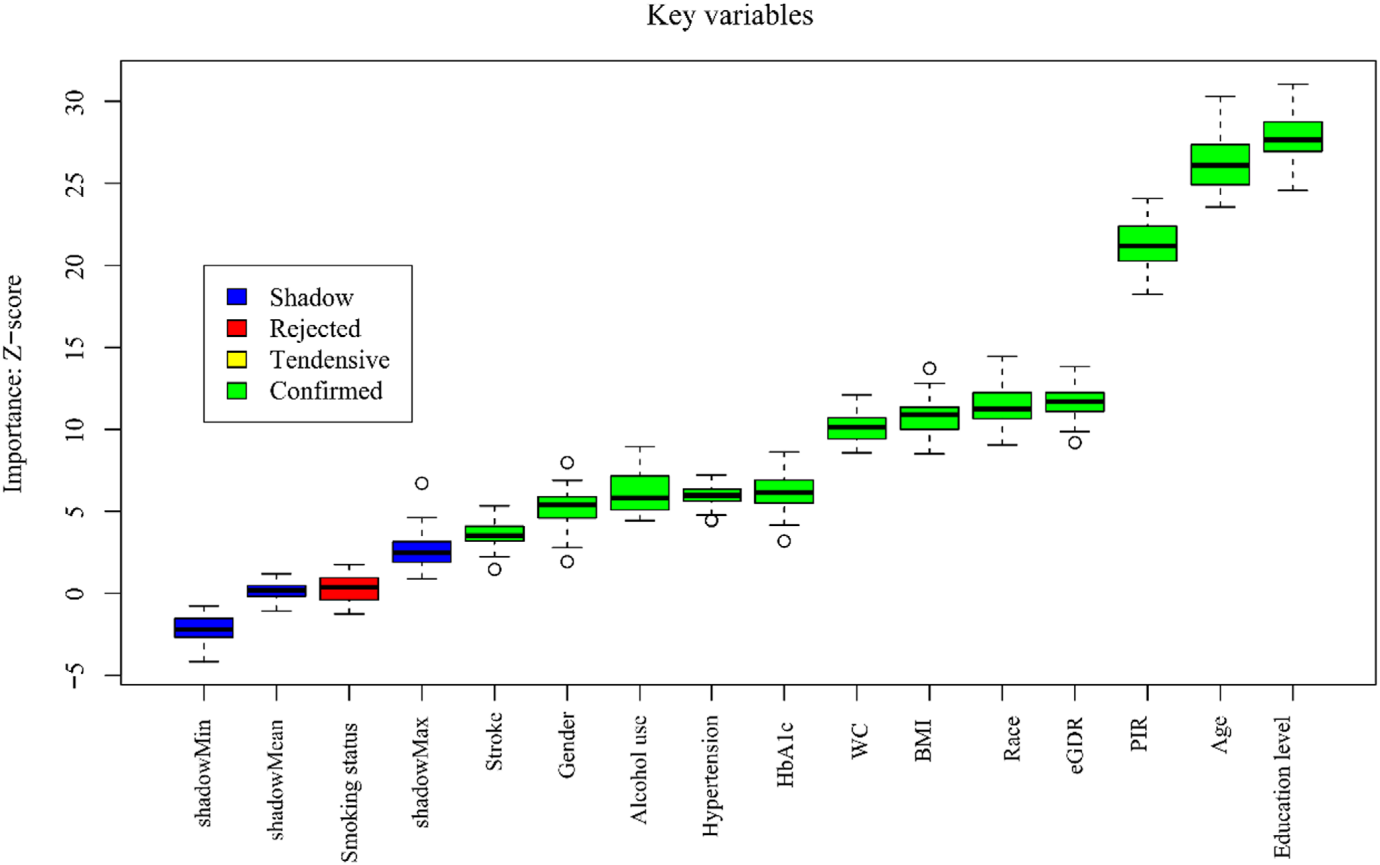
Boruta algorithm ranking of key risk factors for cognitive impairment.

In the Boruta algorithm analysis, variables identified in the green zone, including eGDR, were confirmed as significant risk factors, underscoring their critical role in cognitive impairment. Specifically, eGDR emerged as a key risk factor, with its elevated *Z*‐score indicating a substantial contribution to the overall risk profile. In contrast, variables in the red zone, such as smoking status, were classified as non‐significant, reflecting their negligible association with the risk of cognitive impairment. For further details, refer to Figure 4.

### 3.5 Development and Validation of a Machine Learning Prediction Model for eGDR and Cognitive Impairment

**FIGURE 5:**
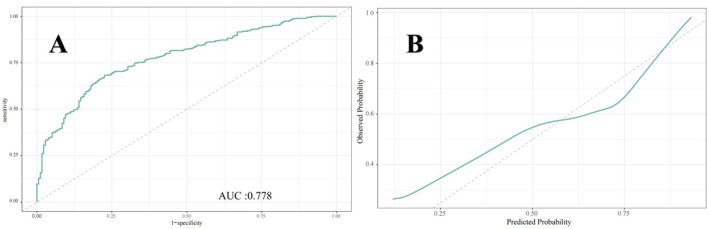
(A) ROC and (B) calibration curves of the LASSO model for evaluating eGDR and cognitive impairment.

**FIGURE 6:**
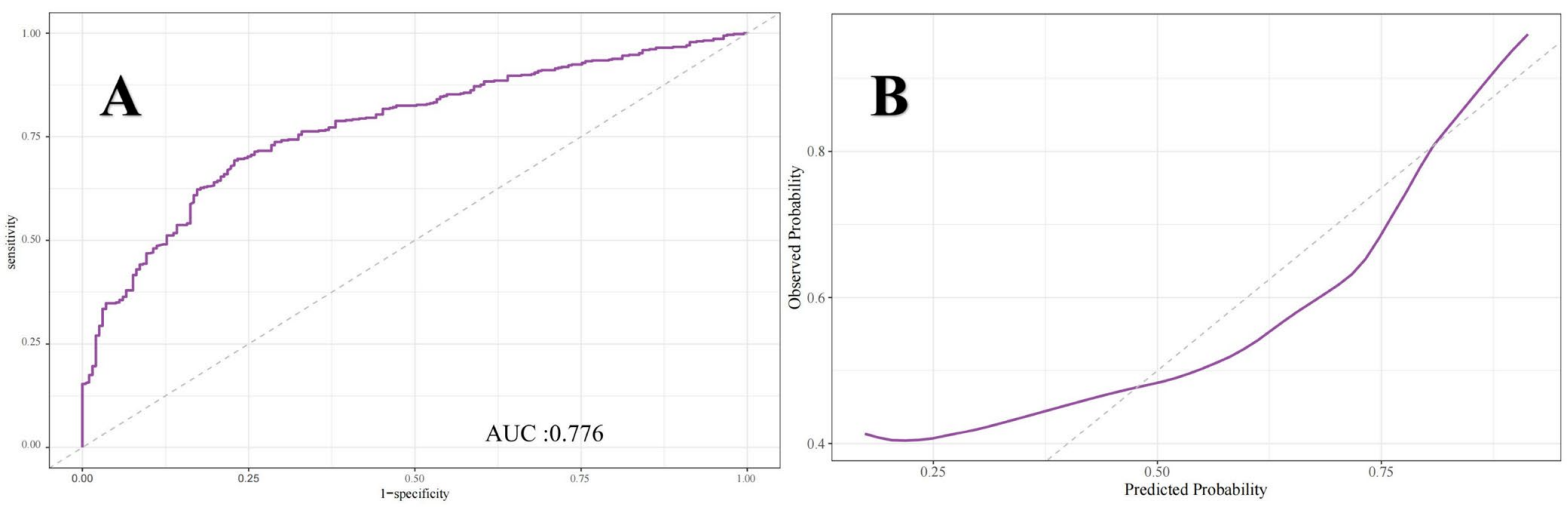
(A) ROC and (B) calibration curves of the XGBoost model for evaluating eGDR and cognitive impairment.

**FIGURE 7:**
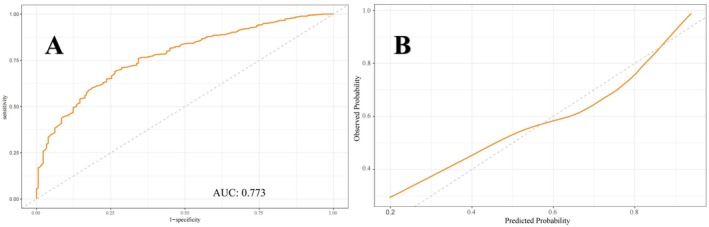
(A) ROC and (B) calibration curves of the Random Forest model for evaluating eGDR and cognitive impairment.

**FIGURE 8:**
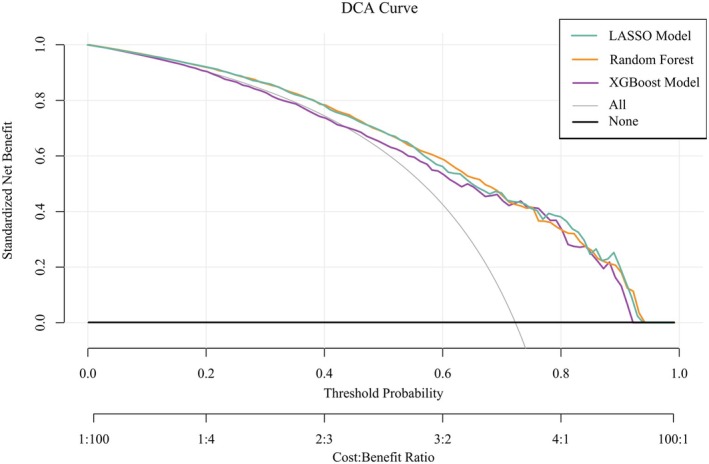
Net benefit comparison of LASSO, Random Forest, and XGBoost Models in clinical decision‐making for eGDR and cognitive impairment.

Figures 5, 6, 7 present the ROC curves (Figures 5, 6, 7) and corresponding calibration plots (Figures 5, 6, 7) for the LASSO, XGBoost, and Random Forest models in relation to eGDR and cognitive impairment. LASSO was chosen for its capacity to handle high‐dimensional data through variable selection, thereby improving model interpretability and reducing the risk of overfitting. XGBoost was selected for its robust predictive performance, particularly its ability to model complex relationships and interactions within the data, as well as its capability to manage missing data and apply regularization to mitigate overfitting. Random Forest was employed due to its ensemble approach, which combines multiple decision trees to minimize variance and enhance the accuracy of predictions, making it highly effective for both categorical and continuous variables. The results indicate that all three machine learning models exhibit ROC curves with an AUC greater than 0.7, and their calibration plots confirm successful model development. Among these, the LASSO model performed the best, with an AUC of 0.778 (Figure 5A), while the Random Forest model demonstrated the most favorable calibration results (Figure 7B). Furthermore, the DCA shown in Figure 8 reveals that the net benefit curves for LASSO, Random Forest, and XGBoost are relatively similar and significantly above the “None” and “All” reference lines. This suggests that these models provide considerable net benefits for clinical decision‐making regarding eGDR and cognitive impairment across different threshold probabilities, thereby offering effective decision support.

### 3.6 Comparison of eGDR With Other IR Indices (TyG, TG/HDL‐C, METS‐IR) for Predicting Cognitive Impairment

To further analyze the predictive performance of eGDR, TyG, TG/HDL‐C, and METS‐IR for cognitive impairment, participants with incomplete data on FPG, TG, and HDL‐C were excluded to ensure the accuracy of the results. This led to a final sample of 1155 participants. Cognitive impairment was defined based on cognitive scores, with the 25th percentile value (*P*_25_ = −1.77) used as the threshold to classify participants into cognitive impairment and non‐cognitive impairment groups. Table S2 provides a detailed comparison of demographic characteristics and other relevant variables between the two groups of participants.

**FIGURE 9:**
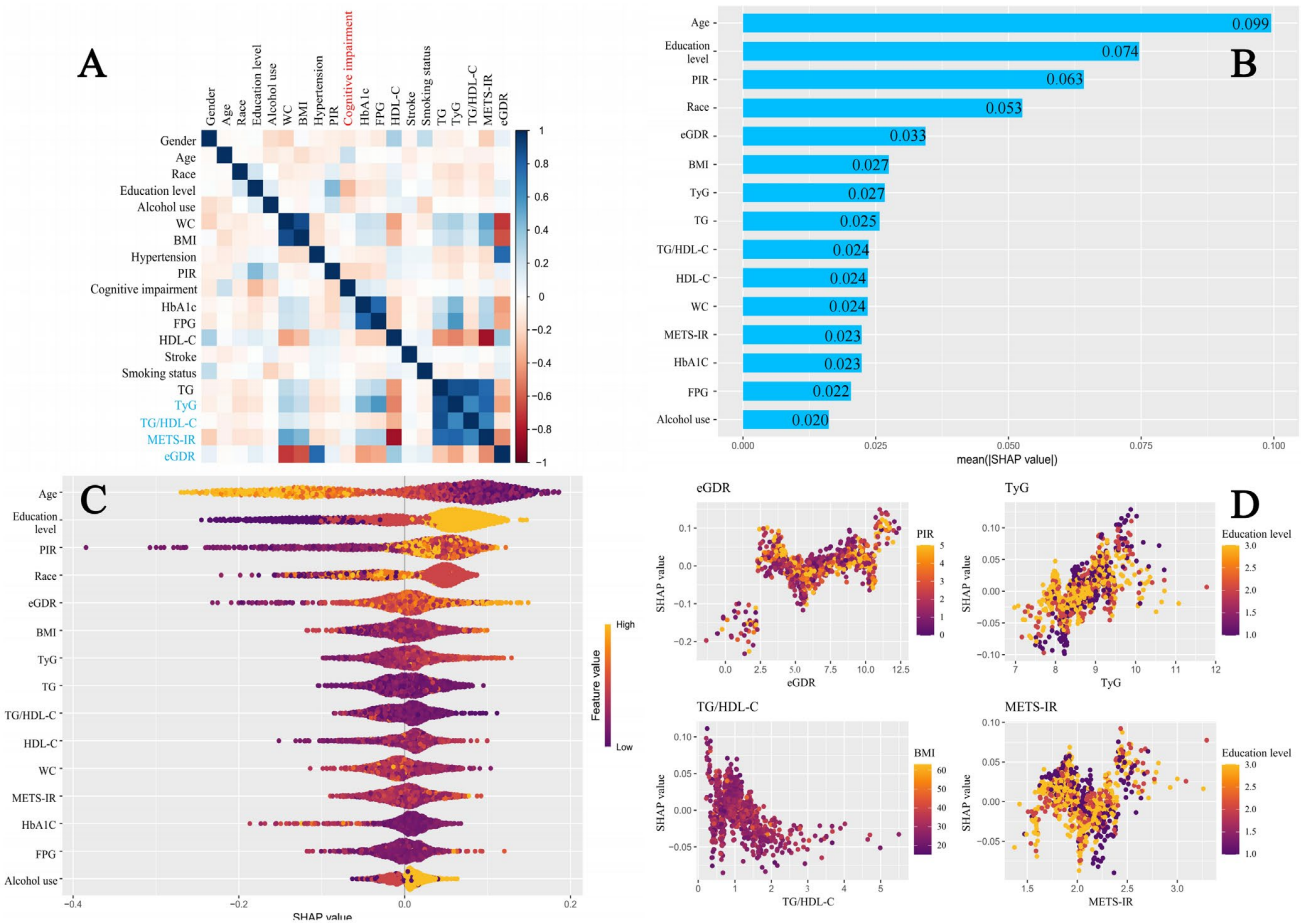
(A) Correlation analysis of IR indicators and related factors in cognitive impairment. (B) Feature importance assessment of study variables in cognitive impairment using SHAP values. (C) SHAP value distribution of study variables in relation to cognitive impairment. (D) SHAP dependence plots for eGDR, TyG, TG/HDL‐C, and METS‐IR.

Through correlation analysis and SHAP modeling, the relationship between the study variables and cognitive impairment was further explored. Figure 9A presents the correlation analysis between the study variables and cognitive impairment. Among the IR surrogate markers examined, eGDR and TG/HDL‐C showed a negative correlation with cognitive impairment, while TyG and METS‐IR were positively correlated. The strongest correlation was observed for eGDR. Figure 9B,C show the variable importance for predicting cognitive impairment based on SHAP values. The *x*‐axis in Figure 9B (mean (|SHAP value|)) represents the average absolute SHAP value for each variable, indicating its relative importance in predicting cognitive impairment. The results highlight that, compared to other IR surrogate markers, eGDR holds the highest predictive importance. Figure 9D illustrates a positive correlation between the SHAP values for eGDR, TyG, and METS‐IR, except for TG/HDL‐C, with eGDR showing the strongest relationship.

## 4 Discussion

This study systematically examined the relationship between eGDR and cognitive function, revealing that higher eGDR levels are significantly associated with improved cognitive scores and a reduced risk of cognitive impairment. Using multivariable linear and logistic regression analyses, we found that for each unit increase in eGDR, cognitive scores increased by 0.095 points, while the likelihood of cognitive dysfunction decreased by 7.5%. Quartile analysis further supported these findings, with individuals in Q4 showing a 0.541‐point improvement in cognitive scores and a 32.8% reduction in the risk of cognitive impairment compared to those in Q1. Interaction tests confirmed the robustness of these associations across various population and clinical subgroups, with no significant interactions detected. These results underscore the potential of eGDR as a reliable predictor of cognitive health status across diverse populations. Additionally, RCS analysis demonstrated a significant inverse linear relationship between eGDR and the risk of cognitive impairment, supporting the hypothesis that higher eGDR levels may serve as a protective factor against cognitive decline. Machine learning models, including LASSO, XGBoost, and random forests, validated the feasibility of using eGDR to predict cognitive dysfunction, as confirmed by ROC curve analysis. DCA further reinforced the feasibility of intervening on eGDR levels to safeguard cognitive health. Correlation analysis and SHAP modeling comparing eGDR with other IR indices (TyG, TG/HDL‐C, METS‐IR) confirmed that eGDR is a more significant factor influencing cognitive impairment.

A cohort study conducted in China found that higher eGDR levels were associated with slower cognitive decline, with a more pronounced effect observed in women [45]. In contrast to this study, our research not only confirms the protective effect of eGDR on cognitive function, but also demonstrates that, compared to other IR indices (such as TyG, TG/HDL‐C, and METS‐IR), eGDR plays a more pivotal role in cognitive outcomes. IR is thought to impair cognitive function through several mechanisms, including the promotion of β‐amyloid deposition and tau protein hyperphosphorylation, inhibition of hippocampal synaptic plasticity, induction of neuroinflammation, disruption of lipid metabolism, and impaired insulin transport across the blood–brain barrier, leading to neuronal metabolic dysfunction and damage [17]. As a proxy for IR, eGDR incorporates WC, HbA1c, and hypertension—three components that independently contribute to cognitive function through inflammation, oxidative stress, and cerebrovascular pathology.

WC is a key indicator of visceral fat accumulation and is more strongly associated with metabolic syndrome components and health risks than other anthropometric measures such as BMI or fat percentage [46, 47]. Visceral fat secretes pro‐inflammatory cytokines, including interleukin‐6 (IL‐6) and tumor necrosis factor‐α (TNF‐α), which stimulate hepatic secretion of C‐reactive protein (CRP), leading to systemic low‐grade inflammation [48]. CRP and IL‐6 negatively impact cognitive function through distinct mechanisms: CRP is linked to reduced gray matter volume and increased blood–brain barrier permeability, while IL‐6 impairs neural responses and neuroplasticity, affecting information processing speed and attentional control [49]. Furthermore, chronic low‐grade inflammation caused by visceral fat triggers oxidative stress, impairing endothelial function and reducing nitric oxide (NO) production, which disrupts central nervous system blood flow regulation and negatively affects brain function [50].

HbA1c, a measure of long‐term glycemic control, is linearly associated with cognitive decline, particularly in memory and executive function [51, 52]. Hyperglycemia induces oxidative stress and advanced glycation end products (AGEs), which disrupt mitochondrial and endothelial cell function, exacerbating neuronal and white matter damage [53, 54]. AGEs further activate neuroinflammatory pathways, such as the AGEs‐RAGE signaling cascade, releasing inflammatory cytokines and reactive oxygen species (ROS) that accelerate neurodegeneration [55]. Moreover, elevated HbA1c levels are associated with dyslipidemia, characterized by increased LDL cholesterol and TG and reduced HDL‐C, which exacerbate atherosclerosis and vascular dysfunction, heightening the risk of cerebrovascular disease and brain aging [56]. These findings suggest that maintaining optimal glycemic control may help mitigate cognitive impairment and brain damage.

Hypertension is a well‐established risk factor for cognitive decline, associated with white matter lesions, cerebral microbleeds, brain atrophy, and ischemic stroke [57]. Uncontrolled hypertension can impair cognitive function through multiple pathways. Hypertension increases the risk of cerebral microvascular damage and infarction due to arteriosclerosis, lipohyalinosis, endothelial dysfunction, and impaired cerebral autoregulation, resulting in reduced cerebral perfusion, white matter lesions, and localized ischemia [58]. Additionally, hypertension‐induced oxidative stress and neuroinflammation activate microglia and perivascular macrophages, exacerbating white matter damage and neuronal injury [59]. Hypertension also disrupts the integrity of the blood–brain barrier, allowing toxic molecules, cells, and inflammatory factors to infiltrate the brain, further impairing cerebral blood flow regulation, white matter integrity, and neuronal function, ultimately accelerating neurodegeneration [60]. These findings underscore the necessity of effective blood pressure control for preserving cognitive health [61].

This study provides important insights into the relationship between eGDR and cognitive function, but several limitations should be acknowledged. First, the cross‐sectional design limits the ability to establish causality. While the findings suggest a protective role of eGDR in cognitive health, longitudinal cohort studies and interventional trials are needed to confirm these associations and clarify causation. Second, the study population was predominantly drawn from specific geographic, racial, and cultural backgrounds, which may limit the generalizability of the findings. Given that the sample is primarily from the United States, the results may not be fully applicable to other regions or populations with different racial and cultural characteristics. Future studies should validate these findings in diverse ethnic, cultural, and geographic groups to enhance external validity. Additionally, the discussion should address other potential confounding factors, such as mental health, lifestyle factors (e.g., diet, physical activity), or medication use, which may play a role in cognitive impairment, especially during early screening stages.

This study lays a foundation for exploring the clinical and public health significance of eGDR as a predictive marker for cognitive impairment. To enhance the generalizability and external validity of these findings, future research should include large‐scale, multicenter prospective studies across various populations and clinical settings. Mechanistic studies are needed to clarify the biological pathways linking eGDR to cognitive health, focusing on IR‐related processes such as neuroinflammation, oxidative stress, endothelial dysfunction, and blood–brain barrier disruption. Additionally, combining eGDR with other biomarkers, such as neuroimaging indicators (e.g., hippocampal volume, amyloid deposition) and genetic risk factors (e.g., ApoE genotype), could refine risk prediction models and improve accuracy. Finally, intervention studies targeting modifiable eGDR components—such as glycemic control, blood pressure management, and central obesity reduction—should evaluate whether improving eGDR levels can prevent cognitive decline or reduce the incidence of cognitive impairment. These efforts could guide tailored prevention and treatment strategies, ultimately alleviating the burden of cognitive disorders in aging populations.

## 5 Conclusion

This study provides compelling evidence that elevated eGDR is associated with improved cognitive function and a reduced risk of cognitive impairment. After adjusting for potential confounders, we found that each unit increase in eGDR was associated with a significant increase in cognitive scores and a decrease in the likelihood of cognitive dysfunction. Furthermore, quartile analysis demonstrated that the highest eGDR quartile was associated with better cognitive scores and reduced risk of cognitive impairment. These findings were robust across various subgroups and clinical conditions, emphasizing the potential of eGDR as a reliable predictor of cognitive health. The analysis also highlighted that higher eGDR levels may act as a protective factor against cognitive decline, a conclusion further supported by RCS analysis. Machine learning models, including LASSO, XGBoost, and Random Forest, validated eGDR's predictive capability for cognitive impairment. Additionally, eGDR was found to be a stronger predictor of cognitive impairment than other IR indices, underscoring its importance in clinical decision‐making. Overall, these findings suggest that monitoring and managing eGDR could be an effective strategy for safeguarding cognitive health, particularly in at‐risk populations.

## Author Contributions

**Tianyi Wang:** conceptualization, data curation, formal analysis, investigation, resources, software, writing – original draft, writing – review and editing. **Haochen Jiang:** data curation, formal analysis, investigation, software, writing – original draft, writing – review and editing. **Ruwen Zheng:** investigation, methodology, project administration, writing – review and editing. **Chuchu Zhang:** methodology, project administration, writing – review and editing. **Xiumei Ma:** project administration, validation, resources, visualization, writing – review and editing. **Yi Liu:** conceptualization, data curation, funding acquisition, project administration, resources, validation, writing – original draft, writing – review and editing.

## Ethics Statement

This study protocol was reviewed and approved by the research ethics review board of the U.S. Centers for Disease Control and the National Center for Health Statistics. All participants in NHANES provided written informed consent to participate. The approval numbers for the surveys presented are: Protocol #2011‐17 (NHANES 2011‐2012), Continuation of Protocol #2011‐17 (NHANES 2013‐2014). More information about the NHANES database can be found at https://www.cdc.gov/nchs/nhanes/irba98.htm.

## Consent

All authors read and approved the manuscript for publication.

## Conflicts of Interest

The authors declare no conflicts of interest.

## Supporting information


Tables S1–S2.

## Data Availability

The original contributions presented in the study are included in the article/Supporting Information; further inquiries can be directed to the corresponding authors.
