# Supplementary material for: Assessing the Impact of Estimated Glucose Disposal Rate (eGDR) on Cognitive Function in Older Adults: A NHANES‐Based Machine Learning Study
Source: CNS Neurosci Ther. 2025 Jul 14;31(7):e70524. doi: 10.1111/cns.70524 (PMC12260214; doi:10.1111/cns.70524)
Supplement: Supplementary file 1 — Tables S1–S2. [file CNS-31-e70524-s001.docx]

| Table S1  Normality Test Results and Statistical Methods for Continuous Variables Across Groups | | | | |
| --- | --- | --- | --- | --- |
| Variable | Group | Shapiro-Wilk Test | p-value (SW Test) | Statistical Test Applied |
| WC | Q1 | 0.965 | 0 | Kruskal-Wallis |
|  | Q2 | 0.868 | 0 |  |
|  | Q3 | 0.957 | 0 |  |
|  | Q4 | 0.967 | 0 |  |
| HbA1c | Q1 | 0.79 | 0 | Kruskal-Wallis |
|  | Q2 | 0.708 | 0 |  |
|  | Q3 | 0.785 | 0 |  |
|  | Q4 | 0.848 | 0 |  |
| CERAD | Q1 | 0.988 | 0 | Kruskal-Wallis |
|  | Q2 | 0.988 | 0 |  |
|  | Q3 | 0.983 | 0 |  |
|  | Q4 | 0.976 | 0 |  |
| AFT | Q1 | 0.982 | 0 | Kruskal-Wallis |
|  | Q2 | 0.972 | 0 |  |
|  | Q3 | 0.979 | 0 |  |
|  | Q4 | 0.989 | 0 |  |
| DSST | Q1 | 0.997 | 0.225 | Kruskal-Wallis |
|  | Q2 | 0.994 | 0.027 |  |
|  | Q3 | 0.997 | 0.295 |  |
|  | Q4 | 0.996 | 0.187 |  |
| Cognitive scores | Q1 | 0.998 | 0.688 | ANOVA |
|  | Q2 | 0.995 | 0.057 |  |
|  | Q3 | 0.998 | 0.863 |  |
|  | Q4 | 0.996 | 0.092 |  |

| Table S2  Baseline Characteristics of Participants (N=1155) Stratified by Cognitive Impairment Status | | | |
| --- | --- | --- | --- |
|  | Cognitive Impairment Status | |  |
| Characteristic | With Cognitive Impairment | Without Cognitive Impairment | *P* |
| N | 289 | 866 |  |
| Age (years)(%) |  |  | <0.001 |
| <70 | 106 (36.68%) | 523 (60.39%) |  |
| ≥70 | 183 (63.32%) | 343 (39.61%) |  |
| Gender (%) |  |  | 0.026 |
| Male | 158 (54.67%) | 408 (47.11%) |  |
| Female | 131 (45.33%) | 458 (52.89%) |  |
| Race (%) |  |  | <0.001 |
| Mexican American | 39 (13.49%) | 63 (7.27%) | \|  \| \| --- \| |
| Other Hispanic | 45 (15.57%) | 68 (7.85%) |  |
| Non-Hispanic White | 111 (38.41%) | 492 (56.81%) |  |
| Non-Hispanic Black | 76 (26.30%) | 154 (17.78%) |  |
| Other Race | 18 (6.23%) | 89 (10.28%) |  |
| Education level (%) |  |  | <0.001 |
| Below high school | 119 (41.18%) | 134 (15.47%) |  |
| High school or GED | 84 (29.07%) | 196 (22.63%) |  |
| Above high school | 86 (29.76%) | 536 (61.89%) |  |
| Alcohol use(%) |  |  | <0.001 |
| Never | 58 (20.07%) | 120 (13.86%) |  |
| Former | 106 (36.68%) | 210 (24.25%) |  |
| Now | 125 (43.25%) | 536 (61.89%) |  |
| Smoking status(%) |  |  | 0.671 |
| Never | 141 (48.79%) | 435 (50.23%) |  |
| Now | 148 (51.21%) | 431 (49.77%) |  |
| Stroke (%) |  |  | 0.266 |
| Yes | 25 (8.65%) | 58 (6.70%) |  |
| No | 264 (91.35%) | 808 (93.30%) |  |
| Hypertension (%) |  |  | 0.006 |
| Yes | 200 (69.20%) | 521 (60.16%) |  |
| No | 89 (30.80%) | 345 (39.84%) |  |
| BMI (%) |  |  | 0.475 |
| Normal | 69 (23.88%) | 238 (27.48%) |  |
| Overweight | 104 (35.99%) | 302 (34.87%) |  |
| Obese | 116 (40.14%) | 326 (37.64%) |  |
| WC (cm) | 103.58 ± 14.71 | 101.99 ± 15.14 | 0.118 |
| PIR | 2.04 ± 1.47 | 2.86 ± 1.56 | <0.001 |
| HbA1c (%) | 6.31 ± 1.34 | 5.98 ± 0.97 | <0.001 |
| CERAD | 18.21 ± 5.07 | 27.62 ± 4.90 | <0.001 |
| AFT | 11.81 ± 3.48 | 18.66 ± 4.98 | <0.001 |
| DSST | 28.54 ± 10.70 | 52.39 ± 14.56 | <0.001 |
| Cognitive scores | -3.06 ± 1.01 | 1.02 ± 1.76 | <0.001 |
| FPG (mg/dL) | 120.93 ± 46.10 | 113.02 ± 30.38 | 0.045 |
| HDL-C (mg/dL) | 53.29 ± 17.28 | 56.30 ± 16.44 | <0.001 |
| TG (mg/dL) | 124.59 ± 74.33 | 122.63 ± 72.58 | 0.505 |
| eGDR | 8.74 ± 0.66 | 8.68 ± 0.60 | 0.206 |
| TyG | 1.07 ± 0.56 | 1.11 ± 0.64 | 0.537 |
| TG/HDL-C | 2.08 ± 0.26 | 2.05 ± 0.26 | 0.007 |
| METS-IR | 6.00 ± 2.44 | 6.64 ± 2.52 | <0.001 |
| Mean ± SD for continuous variable，number (%) for categorical variables. | | | |
